# Advantages of patient-specific cutting guides with disposable instrumentation in total knee arthroplasty: a case control study

**DOI:** 10.1186/s13018-021-02310-y

**Published:** 2021-03-15

**Authors:** Kevin Moerenhout, Behrang Allami, Georgios Gkagkalis, Olivier Guyen, Brigitte M. Jolles

**Affiliations:** 1 Department of Orthopaedics and Traumatology, Lausanne University Hospital and University of Lausanne (UNIL), Lausanne, Switzerland; 2 Department of Muskuloskeletal Medicine, Swiss BioMotion Lab, Lausanne University Hospital and University of Lausanne (UNIL), Lausanne, Switzerland; 3 Ecole Polytechnique Fédérale de Lausanne (EPFL), Institute of Microengineering, Lausanne, Switzerland

**Keywords:** Total knee arthroplasty, Patient-specific instrumentation, Patient-specific cutting guide, Disposable instrumentation, Single-use, Operative time, Sterilisation cost

## Abstract

**Background:**

Total knee arthroplasty (TKA) is most frequently planned using conventional two-dimensional weight-bearing lower limb radiographs and is performed with conventional femoral and tibial cutting guides. Questions have been raised about the accuracy of conventional TKA instrumentation and planning for an anatomically standard or complex joint. Use of computed tomography (CT)-based three-dimensional (3D) templating and patient-specific cutting guides printed in 3D has shown improved postoperative lower limb alignment parameters. This case-control study compared costs and operative times of using CT-based, patient-specific, single-use instruments versus conventional metal instruments for TKA.

**Methods:**

In this case-control, retrospective chart review, all TKAs were performed by one senior surgeon, using the F.I.R.S.T. posterior-stabilised knee prosthesis (Symbios, CH), with a similar protocol and identical operating room setup. Group A included 51 TKAs performed with patient-specific cutting guides and conventional metal instruments. Group B included 49 TKAs performed with patient-specific cutting guides and patient-specific, single-use instrumentation. Operation duration, number of instrumentation trays and sterilisation costs were evaluated.

**Results:**

The groups were similar for age, body mass index, hip-knee-ankle angle and operation duration. The mean number of instrumentation trays was 8.0 ± 0.8 for group A (controls) and 5.1 ± 0.9 for group B (*p*<0.001). The mean sterilisation costs were 380 ± 47 Swiss Francs (CHF) for group A and 243 ± 55 CHF for group B (*p*<0.001), for a mean cost reduction of 130.50 CHF per intervention in group B. The time interval between two consecutive surgeries was 24 min for group A and 18 min for group B. There were no adverse events or complications, instrument-related or otherwise.

**Conclusion:**

Compared to conventional instrumentation, use of patient-specific, single-use instruments for TKA reduced the number of instrumentation trays by more than one-third and saved 36% in sterilisation costs. If fabrication costs of single-use instruments are included by the company, the total cost is significantly diminished. There was no operative time advantage for single-use instrumentation.

## Background

Total knee arthroplasty (TKA) has become a routine procedure in the last decade. The incidence of primary TKA in the USA is estimated to increase between 143 and 855% by 2050 compared to 2012 [1]. In current practice, surgery is most frequently planned using conventional weight-bearing lower limb radiographs and is performed with conventional femoral and tibial cutting guides. Questions have been raised about the accuracy of conventional TKA instrumentation and two-dimensional planning for an anatomically standard or complex joint [2, 3].

Computer tomography (CT)-based three-dimensional (3D) templating and patient-specific cutting guides are an innovative alternative to conventional cutting guides for TKA. Several studies have shown improved postoperative lower limb alignment parameters, such as hip-knee-ankle (HKA) angle [4, 5] and alpha and beta angles [6], improved accuracy of implant size determination and positioning of tibial implant [7] and favourable femoral rotational alignment [8] with CT-based, 3D, patient-specific instrumentation compared to conventional instruments. Whether mechanical alignment outliers are reduced using patient-specific guides and instrumentation remains controversial [4, 5, 9–12]; some studies reported shorter operating time with the use of patient-specific instrumentation [10, 13, 14], others did not [15, 16].

As these patient-specific CT-based guides are printed in 3D, it is also possible to create customised, single-use surgical instruments dedicated to the patient. This could theoretically simplify the operative procedure for the surgeon, as fewer instruments would be needed to prepare the femur and knee for the implants. Only one sterilised box of materials would be needed, less than the usual five to seven conventional instrumentation trays. A combination of customised, patient-specific cutting guides and customised, disposable, 3D-printed instrumentation could be more cost-effective than patient-specific guides with conventional metal instruments. This technique is now available with a full set of personalised, single-use instruments, substantially replacing the conventional instrumentation trays.

The aim of this study was to evaluate the costs and surgical duration when using a customised, disposable instrumentation set compared to conventional instrumentation for TKA performed with patient-specific guides.

## Methods

A case-control study design was utilised. The charts of patients who underwent primary TKA for primary knee osteoarthritis were retrospectively reviewed. Exclusion criteria were a body mass index (BMI) ≥35kg/m^2^, deformity in the frontal plane >10°, previous knee operation, the necessity of a lateral parapatellar approach and perioperative complications due to something other than the instrumentation. This study was approved by the institution’s Research Ethics Board (CER-VD 278/10).

**Fig. 1:**
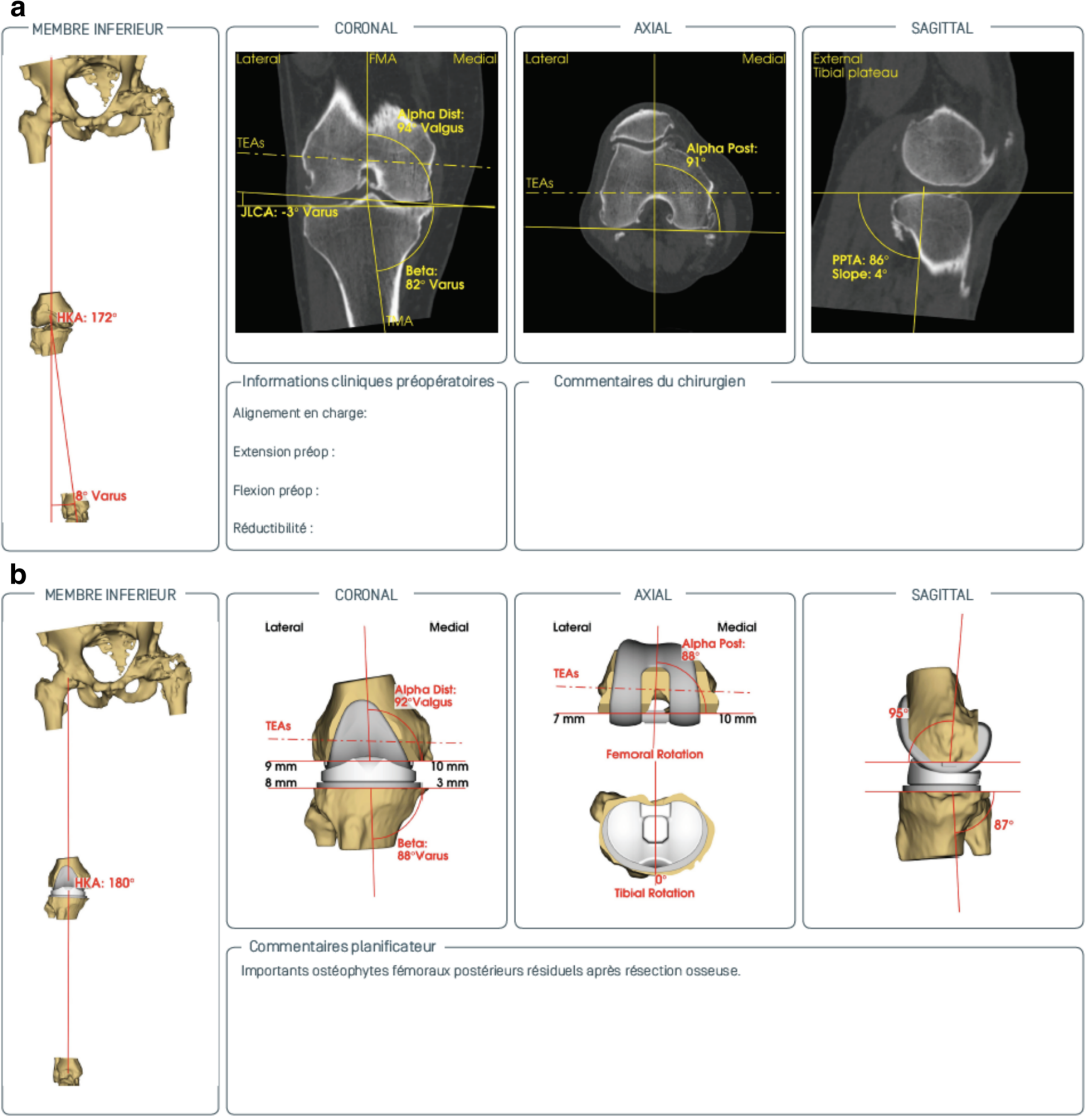
CT-based three-dimensional preoperative planning with **a** analysis of the different angles and **b** planning of femoral and tibial prosthetic implant for anatomic reconstruction

Prior to surgery, all patients underwent CT imaging required for custom manufacture of patient-specific cutting guides (Knee-Plan® Symbios, CH) (Fig. 1). All TKAs were performed by one senior surgeon, using the F.I.R.S.T. posterior-stabilised knee prosthesis (Symbios, CH), with a similar protocol and identical operating room setup.

**Fig. 2:**
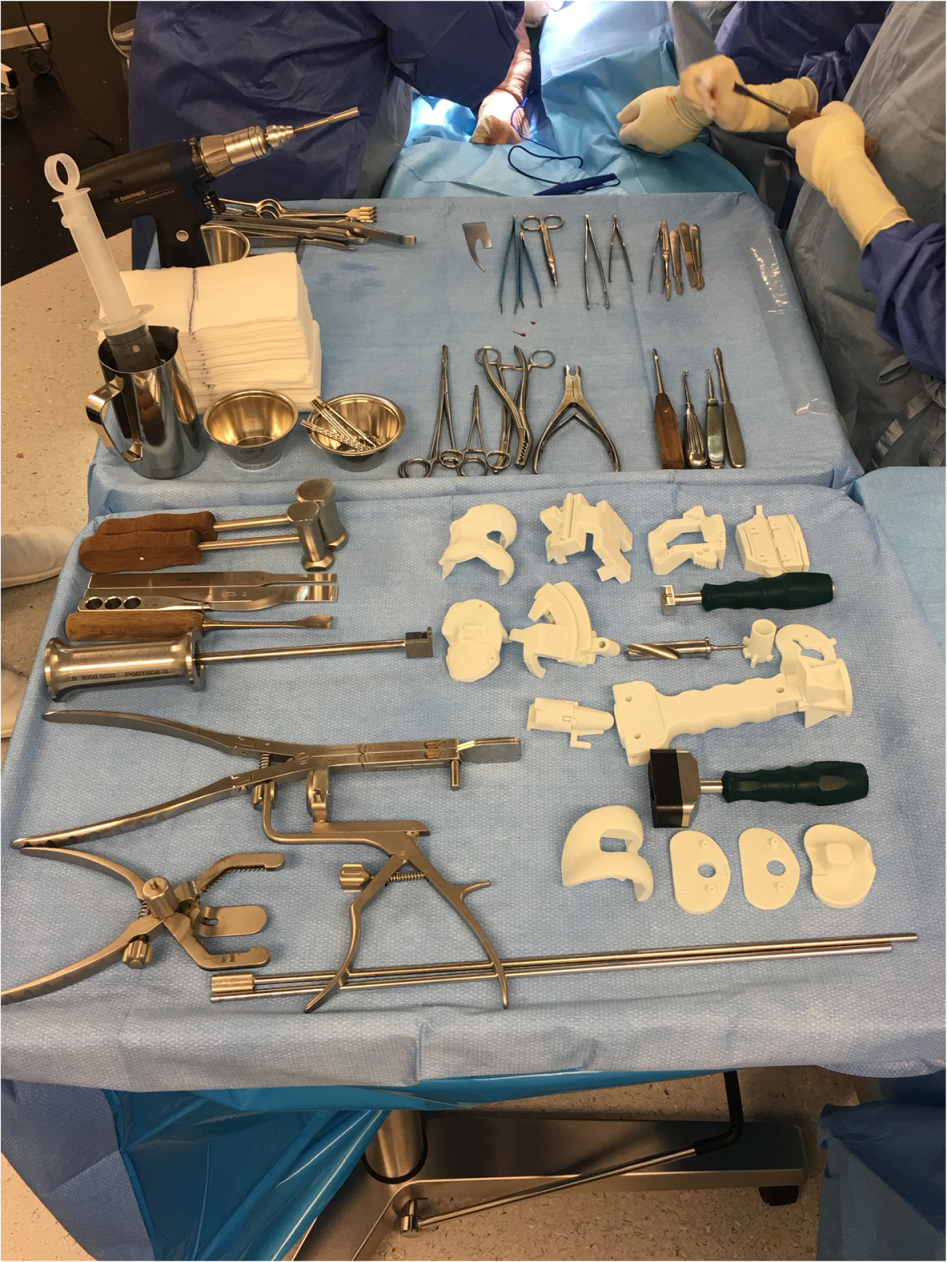
Two surgical instrument trays in the operating room. The upper tray contains standard metal instruments. The lower tray contains standard metal instruments and the different CT-based, patient-specific, single-use instruments, ancillary and cutting guides (Knee-Plan® Set), shown in white. Single-use material in first (top) row, from left to right: femoral model, distal femoral cutting guide, intercondylar cutting block, 4-in-1 cutting block. Second row: tibial model, tibial cutting guide, tibial drill guide, tibial trial. Third row: tibial stem trial, impactor with impaction pad. Fourth (bottom) row: femur trial, 2 trial insert augments (+2mm and +5mm), trial insert

The control group (group A) consisted of 51 consecutive TKAs performed with patient-specific cutting guides (Knee-Plan® Guides, Symbios, CH) and conventional instrumentation. The case group (group B) consisted of 49 consecutive TKAs performed with patient-specific cutting guides (same Knee-Plan® Guides) and customised, patient-specific, disposable instrumentation (Knee-Plan® Set, Symbios, CH) (Fig. 2). All patient-specific guides were manufactured based on a CT scan after validation by a senior surgeon.

The following parameters were assessed for the two groups: duration of operation, number of instrumentation trays used and sterilisation costs. Sterilisation costs were calculated by multiplying the number of trays needed by the mean sterilisation cost of 43.50 Swiss franc (CHF) per tray. The time interval between two consecutive surgeries was also recorded.

### Statistical analysis

Separate sample size calculations for the parameters of duration of operation, number of trays and sterilisation costs found duration of operation required the largest sample size. A minimum of 35 patients in each cohort was determined to be necessary to detect a significant difference in operation duration, with a power of 80% and a threshold of 5%.

Statistical analysis was performed using the XLSTAT statistical software (Addinsoft®, Paris, France). An unpaired *t* test was used for normally distributed continuous variables, and the Mann-Whitney *U* test was used for variables that were not normally distributed. A *p* value <0.05 was considered statistically significant.

## Results

The two groups were similar for age, BMI and HKA angle (Table 1). Operation duration was similar in both groups (Table 1).

**Table 1:** Patient demographics and operative parameters (shown as mean, with standard deviation in brackets) for control group with standard metal instrumentation (group A) and for group with patient-specific, single use instrumentation (group B) for total knee arthroplasty. Patient-specific cutting guides were used in both groups

	Group A (control) (*n*=51)	Group B (single-use instrumentation) (*n*=49)	*p* value
*Demographics*			
Gender (male); *n* (%)	21 (41%)	22 (45%)	0.330
Age (years)	71.1 (9.2)	70.2 (9.5)	0.632
BMI (kg/m^2^)	28.6 (3.3)	27.6 (3.6)	0.141
HKA angle (°)	176.7 (4.2)	176.7 (4.3)	0.960
*Operative parameters*			
Operative time (min)	148.2 (16.2)	153.8 (22.8)	0.159
Instrument trays (*n*)	8.0 (0.8)	5.1 (0.9)	<0.001
Sterilisation cost (CHF)	380 (47)	243 (55)	<0.001

*BMI* body mass index; *HKA* hip-knee-ankle; *CHF* Swiss francs

The control group A required significantly more instrumentation trays (mean 8.0) when compared to group B (mean 5.1, *p*<0.001). At a mean sterilisation cost of 43.50 CHF per tray, there was a mean cost reduction of 130.50 CHF per intervention in group B (*p*<0.001) (Table 1).

The time interval between two consecutive surgeries was 24 min for group A and 18 min for group B.

There were no adverse events or complications, instrument-related or otherwise.

## Discussion

Numerous studies have compared TKA using patient-specific instrumentation to TKA performed with conventional instrumentation [4, 5, 10, 11, 13–15]. To the best of our knowledge, none have studied two groups of CT-guided instrumentation using patient-specific cutting guides, one with supplementary personalised, disposable instrumentation, compared to the other with conventional instrumentation.

The current study found a significant difference in the number of instrument trays needed when patient-specific, disposable instruments were used. Disposable instrumentation enabled an average of three fewer trays to be used per case. Fewer instrument trays provide a considerable advantage for both the surgeon and the scrub nurse, for obvious reasons such as material handling and space occupancy. It is important to note that, even with the single-use instrumentation, other basic instrumentation trays with standard material must be utilised (Fig. 2). In a systematic review of the use of patient-specific cutting blocks for TKA, Sassoon et al. also found that fewer trays were needed with patient-specific instrumentation, but they were not able to support its cost-effectiveness [17].

The overall sterilisation cost was significantly reduced with the use of disposable instrumentation, which represents a financial advantage over the sterilisation procedure for the standard instruments. The manufacturing costs for the additional disposable instruments were the same price for all public hospitals, such that the costs of the Knee-Plan® Guides (i.e. patient-specific cutting guides only) and Knee-Plan® Set (i.e. patient-specific cutting guides plus patient-specific, single use instrumentation) were similar. There is also a substantial reduction of storage space required in the operating room with fewer surgical trays, with the advantages of limiting potential loss of material and reducing the risk of compromising sterility (i.e. fewer trays equals less infection risk). However, a conventional instrumentation set must be available, in case it is needed.

As expected, there were no significant differences with respect to operative times. Both groups had CT-based, patient-specific, cutting guides, which is the part of the surgical procedure where one would expect to find time savings. The only parameter that varied was the single-use disposable instrumentation in group B versus the conventional instrumentation used in group A.

The time interval between two consecutive operations was also evaluated and was not significantly different. This parameter is dependent not only on the scrub nurse preparing surgical instrumentation for the next operation, but on many other aspects as well, such as cleaning the operating room and inducing the next patient [18]. Improved operating room efficiency was demonstrated by some studies [19–21], but turnover rates are nuanced [22].

Considering the growing demand of TKA in the next decades [1] and its economic burden [23], any potential cost reductions must be considered. Moreover, if better longevity of implants due to better limb alignment with the use of CT-based patient-specific cutting guides should be confirmed, this could potentially outweigh the costs of supplementary imaging (CT or MRI) and manufacturing of patient-specific guides and instrumentation.

The study has limitations. It is a retrospective, non-randomised study. However, selection bias is unlikely, as both groups were similar for age, BMI and HKA angle, and both groups received patient-specific cutting guides, with a similar surgical protocol. The fact that manufacturing costs for the patient-specific instrumentation were absorbed by the company may represent a bias. The clinical results of the groups were not compared, as this was not the aim of this study. Thus, any potential differences were not identified. A larger, prospective, matched cohort study or randomised controlled trial is needed to ascertain any differences in short-term and long-term clinical outcomes of TKA using patient-specific guides and instrumentation versus traditional instrumentation.

## Conclusion

Compared to conventional instrumentation, the use of patient-specific, single-use instruments for TKA reduced the number of instrumentation trays by more than one-third and enabled a 36% reduction in sterilisation costs. If companies are willing to include the manufacturing costs of the disposable instrumentation within the costs for the patient-specific guides, then this practice can be cost-effective. The results of this preliminary comparative study support the need for larger, prospective, matched cohort studies or multicentre, randomised, controlled trials to further evaluate the cost-effectiveness of disposable instrumentation and, ultimately, to ascertain whether there are any differences in short-term and long-term clinical outcomes of TKA using patient-specific guides and instrumentation versus traditional instrumentation.

## Data Availability

The datasets used and/or analysed during the current study are available from the corresponding author on reasonable request.

## Abbreviations

3D: Three-dimensional
BMI: Body mass index
CH: Switzerland
CHF: Swiss franc
CT: Computed tomography
HKA: Hip-knee-ankle angle
MRI: Magnetic resonance imaging
TKA: Total knee arthroplasty
